# Neurological disorders in Northern Tanzania: A 6-year prospective hospital-based case series

**DOI:** 10.4314/ahs.v22i1.34

**Published:** 2022-03

**Authors:** William P Howlett, Sarah J Urasa, Venance P Maro, Richard W Walker, Kajiru G Kilonzo, Patrick J Howlett, Marieke CJ Dekker

**Affiliations:** 1 Kilimanjaro Christian Medical Centre, Moshi, Kilimanjaro, Tanzania; 2 Centre for International Health University of Bergen, Bergen Norway; 3 Department of Medicine, North Tyneside General Hospital, Rake Lane, North Shields, Tyne, and Wear NE29 8NH; 4 Royal Brompton and Harefield Hospital Trust Fulham Road, London, SW3 6HP

**Keywords:** Tanzania, neurological, disorder, neurological disease, hospital, HIV

## Abstract

**Background:**

The burden of neurological disorders is large and altered by the HIV epidemic.

**Objectives:**

We describe the pattern of neurological disorders and their association with HIV infection in adult patients attending a consultant hospital in Northern Tanzania.

**Methods:**

In this prospective cross-sectional study, we collected data on adult neurological referrals over a 6-year period between 2007–13. The odds of HIV infection, across neurological categories adjusted for age and sex, was calculated.

**Results:**

Of 2037 participants, 54.8% were male and 45.2% were female. The median age of participants was 43 years. The results for HIV screening were available for 992/2037 (48.7%) patients, of whom 306 (30.8%) were seropositive. The most frequent neurological disorders were cerebrovascular disease (19.9%), paraplegia (13.6%), and peripheral neuropathies (8%). Taken together CNS infection accounted for 278/2037 (13.6%). The adjusted odds (aOR) of HIV infection was highest amongst infections; brain abscesses (aOR 107, 95% CI 35.1–470.4) and meningitis/encephalitis (aOR 40.1, 95% CI 13.6–172.9), but also raised in cerebrovascular disease, paraplegia, peripheral neuropathies, cranial nerve palsies, seizures, cerebllar disorders, movement disorders, motor neuron disease and headache.

**Conclusion:**

The main pattern of neurological disorders in Northern Tanzania is presented. The odds of HIV infection was highest in CNS infections and in a wide range of non-communicable neurological disorders.

## Background

The burden of neurological disorders (NDs) globally is large. In 2016 NDs were the second leading cause of death accounting for 9 million or 16.5% of all deaths and the leading cause of disability-adjusted life years (DALYs), accounting for 276 million or 11.6% of all DALYs with stroke, migraine, epilepsy, meningitis and dementia as main causes^1^. In sub-Saharan Africa (SSA) NDs account for almost 30 million DALYs annually with stroke, meningitis, migraine, dementia and epilepsy as main causes^1^, ^2^.

The pattern of NDs in SSA differs from that reported in high income countries, with infections, in particular human immunodeficiency virus (HIV) being more prevalent and neurodegenerative diseases less common^3^–^6^. In SSA NDs typically account for 18–25% of adult medical hospital admissions, the majority of whom either die in hospital or are discharged with disability^3^, ^5^, ^7^. The most common causes of neurological admissions in adults are stroke, infections, paraplegia, neuropathies, coma and seizures^3^–^5^, ^7^. The most common NDs reported in outpatients are seizures/epilepsy, headache, stroke sequelae, neuropathies, movement disorders and neurodegenerative disorders^8^–^11^.

NDs in SSA are also changing over time^1^. This is in part due to an increasing burden of non-communicable diseases (NCDs), reflecting more prevalent cerebrovascular risk factors, rapid urbanization and an increasingly ageing population^12^,^13^. While there has been a decrease in the frequencies of the more traditional central nervous system (CNS) infections including cerebral malaria, tetanus and bacterial meningitis, the increasing burden of HIV, now affecting over 26 million persons in SSA, is significantly altering the pattern of NDs. In 2016 WHO estimated that HIV was the second leading cause of death in SSA and responsible for approximately 720,000 deaths or over 8% of all deaths in the Africa region. HIV affects the nervous system in 3 main ways^14^, ^15^. Firstly, through loss of cellular immunity resulting in CNS opportunistic processes mostly infections, secondly by direct infection resulting in HIV associated neurocognitive dysfunction (HAND), distal sensory neuropathy (DSN) and vacuolar myelopathy (VM) and lastly by autoimmunity resulting in inflammatory related NDs. However less well known are the overall effects of HIV on the existing patterns of NDs in SSA.

In this study we aim to describe the pattern of neurological disorders and specific neurological diseases in a large cohort of adult patients presenting with NDs, either admitted to hospital or attending neurological outpatient clinic and examine their association with HIV status. The information presented is intended to help in understanding the current burden and diversity of NDs in SSA and in particular their relation to HIV.

## Methods

### Patient population

This prospective cross-sectional hospital-based study was conducted in the medical department at Kilimanjaro Christian Medical Centre (KCMC). KCMC is a 600-bed referral consultant teaching hospital located in Moshi in the northern zone of Tanzania with a catchment population of over 16 million people. The study took place over a 6-year period from April 2007 to March 2013. It included both outpatients and inpatients presenting with neurological disorders who attended general medical/neurology outpatient clinic and/or admitted into the medical wards at KCMC under care of a general physician.

According to hospital policy, adult patients were classified as aged >13 years.

### Standard protocol approvals, registrations, and patient consents

Permission to conduct the study was obtained from the head of the medical department and KCMC ethical committee, certificate number 817. Confidentiality was adhered to byusing codes instead of names for identifying patients.

### Data collection

During the study period patients referred for neurology consultation as inpatients or outpatients were examined by the lead author (WH), a consultant neurologist, and a neurological diagnosis was recorded at the time of examination which was either confirmed or amended based on clinical findings and investigations available. The series includes patients who received a formal and complete consultation. It does not include 2-month annual periods during which WH was absent or patients who had brief clinical reviews. Details of participants were recorded in a logbook and were later coded in an electronic database.

A single specific neurological disease diagnosis was made based on clinical, laboratory and radiological findings by WH. On the basis of this, participants were categorized into one of 19 groups of main neurological disorders chosen for their relevance to the study population. In situations where a case may satisfy two groups, for example meningitis/encephalitis and cranial nerve disorder, the disorder relating to the cause of the underlying pathology was given preference. Although in the majority of cases a single clinical neurological disorder was able to be diagnosed, in those cases where this was not possible the patient was categorized into a group describing the main presenting neurological disorder e.g., altered level of consciousness. The definitions used to classify the main neurological disorders are described in detail in Table 1.

**Table 1:** Definitions for main neurological disorders categories

Diagnosis	Definitions
**Cerebrovascular** **disease**	A clinical and/or radiological diagnosis including stroke, ischaemic and haemorrhagic, subarachnoid haemorrhage, and subdural haematoma.
**Infection:** **Meningitis/Encephalitis**	A clinical and/or laboratory/radiological diagnosis which includes cryptococcal, tuberculous, acute bacterial and viral meningitis/encephalitis.
**Infection: Intracerebral** **abscess**	A clinical and/or radiological diagnosis including toxoplasma, pyogenic, tuberculous, and non-differentiated brain abscess.
**Infection: Cerebral** **Malaria/Tetanus/Others**	A clinical and/or laboratory/radiological diagnosis including, cerebral malaria tetanus and other CNS infections.
**Paraplegia**	A clinical and/or radiological/laboratory diagnosis of non-traumatic causes including malignancy, transverse myelitis, Potts disease, syringomyelia, and other causes of paraplegia/quadriplegia of unknown aetiology.
**Peripheral neuropathies**	A clinical and/or laboratory diagnosis of sensory and or motor peripheral neuropathy including Guillain Barré syndrome, subacute combined degeneration and mononeuritis multiplex and others.
**Neuromuscular** **disorders**	A clinical and/or laboratory diagnosis including myasthenia gravis, dermatomyositis, muscular dystrophy, stiff person syndrome and others.
**Cranial nerve palsies**	A clinical diagnosis including facial nerve palsy, optic neuritis, III, IV and V nerve palsy and others.
**Brain tumours**	Include clinically and/or radiologically categorized glioma, meningioma, and pituitary tumour. The general term tumour includes metastatic and diagnostically uncertain tumours.
**Seizure disorders**	Include clinically diagnosed generalized and focal seizure disorders including epilepsy.
**Altered level of** **consciousness**	A clinical diagnosis based on Glasgow Coma Scale and/or disorientation in time, person, or place, and includes acute confusional states, in the absence of alternative clear neurological diagnosis.
**Movement disorders**	A clinical diagnosis including dystonia, catatonia, involuntary movements, chorea, benign essential tremor, and writers' cramp. The movement disorder Parkinson's disease has its own category
**Parkinson's disease**	A clinical diagnosis including Parkinson's disease (PD) and Parkinson plus disorders.
**Cerebellar** **disorders**	A clinical diagnosis categorized as idiopathic and hereditary including Friedreich's ataxia.
**Motor neuron disease**	A clinical diagnosis including amyotrophic lateral sclerosis, progressive muscular atrophy, primary lateral sclerosis, and pseudobulbar palsy.
**CNS** **demyelinating disease**	A clinical and or radiological/laboratory diagnosis including neuromyelitis optica, multiple sclerosis and acute demyelinating encephalomyelitis.
**Dementia**	A clinical diagnosis based on a laboratory/radiological investigations and minimental test score of <24/30 including Alzheimer's, vascular and fronto temporal.
**Headache**	A clinical diagnosis including tension headache, migraine, cluster, idiopathic intracranial hypertension, and others unclassified.
**Functional** **neurological disorders**	A clinical diagnosis including loss of neurological function, psychogenic non-epileptic attacks, movement disorders and others.
**Others**	A generic term in neurological disorder/disease categories that includes patients who were not specifically classified into specific neurological disease categories.

**Figure 2:**
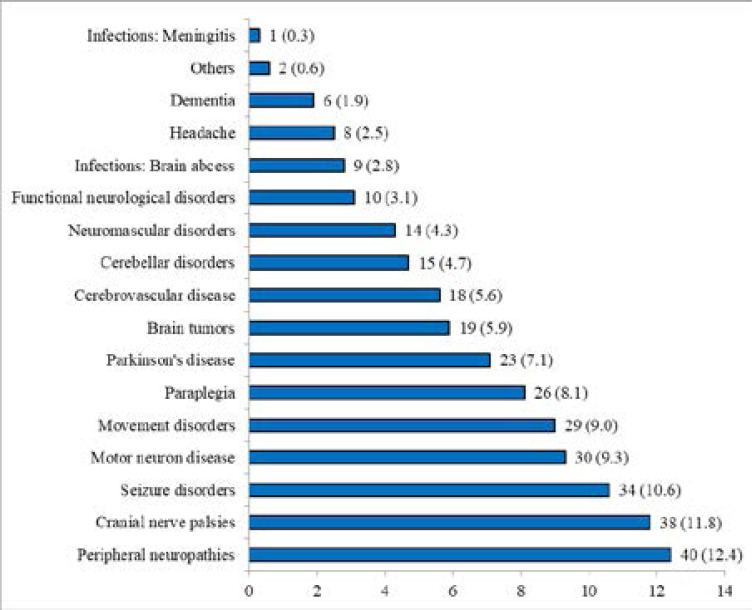
Pattern of main neurological disorders in selected outpatients *(n=332)*

A limitation in the study is that the site of examination was not recorded for all cases. While the majority of cases reported in the series were inpatients the findings in a representative sample of outpatients, n= 332 are presented in Figure 2.

Laboratory and imaging investigations were those carried out routinely at the main laboratory and radiology departments at KCMC. HIV screening was done by two rapid HIV tests (Capillus/Bioline and Determine) and a positive result recorded if both were positive.

Lumbar punctures were carried out as clinically indicated. Results included cell counts, protein and glucose, cryptococcal antigen (CrAg), Gram, ZN and India Ink stains and culture and sensitivities.

Electrophysiological investigations were unavailable at the time of the study. Imaging investigations included Computerized Tomography (CT). A CT scan of head was performed on patients suspected of having an abnormality but was limited due to availability and affordability. The CT scans were interpreted and reported by a qualified radiologist and in cases of diagnostic uncertainty scans were reviewed with WH. Magnetic resonance imaging (MRI) was unavailable at KCMC, although available via referral in exceptional cases. Specialized services including neurosurgery, neuro-oncology, advanced immunodiagnostics, and genetic confirmation were not available during the study period. Data was collected on age, sex, HIV result when performed, and specific neurological disorder/disease diagnostic category.

### Analysis

The frequency and proportions of the specific diagnosis and disorder categories are described according to sex and median age. The results for percentages are rounded to one decimal place. An unadjustedlogistic regression odds ratio of the association between HIV status and diagnosis was calculated. A second a priori adjusted model included age and sex.

A Wald test for significance was calculated for each category. The category brain tumours was used as the baseline category for both models as this was the most well represented group with which HIV was not thought to be markedly associated. In addition, the HIV prevalence (3.6%) in this group was similar to Kilimanjaro regional adult seroprevalence of 3.8% reported in the Tanzanian 2011–12 HIV/AIDS indicator survey. On visual inspection, the age histograms and sex distribution of brain tumours were similar to the whole cohort. To compare those tested and not tested for HIV, a mean age and t-test was used for age, while unadjusted odds ratios and a Chi2 test was used to compare gender. Statistical analysis was performed using R software (version 3.5.1).

## Results

Of 2,038 adult patients referred with a neurological disorder, 1 was missing a final diagnosis and was excluded from further study, while 22 patients with missing data on age and 4 on sex are included in the series. The median age of the study participants (n=2037) was 43 years, (Interquartile range (IQR) 29–58), and 54.8% were male and 45.2% were female. The results for HIV screening were available for 992/2037 (48.7%) patients, of whom 306 (30.8%) were reported seropositive. The frequency and demographics of main neurological disorders are presented in Table 2.

**Table 2:** Frequency and demographics of main neurological disorders (n=2037)

Disorder category	Frequency (%)	*Median age in years (IQR)	*Male (%)	*Female (%)
**Total**	2037	43 (29–58)	1115 (54.8%)	918 (45.2%)
**Cerebrovascular disease**	406 (19.9%)	55 (38.5–68)	252 (62.1%)	154 (37.9%)
**Infection: Intracerebral abscess**	111 (5.4%)	37 (29.5–45)	45 (40.5%)	66 (59.5%)
**Infection: Meningitis/Encephalitis**	110 (5.4%)	33 (25–40)	66 (60%)	44 (40%)
**Infection: Cerebral Malaria/Tetanus/Others**	57 (2.8%)	35 (21–49)	40 (71.4%)	16 (28.6%)
**Paraplegia**	278 (13.6 %)	45 (30–57.25)	165 (59.6%)	112 (40.4%)
**Peripheral neuropathies**	163 (8%)	40 (29–53)	73 (44.8%)	90 (55.2%)
**Brain tumours**	161 (7.9%)	44.5 (31.75–61)	90 (56.25%)	70 (43.75%)
**Seizure disorders**	131 (6.4%)	32.5 (20.25–47.75)	62 (47.3%)	69 (52.7%)
**Cranial nerve palsies**	98 (4.8%)	41 (33–54)	46 (46.9%)	52 (53.1%)
**Movement disorders**	90 (4.4%)	45 (27.25–61.75)	46 (51.1%)	44 (48.9%)
**Altered level of consciousness**	74 (3.6%)	38 (25–52)	53 (71.6%)	21 (28.4%)
**Neuromuscular disorders**	72 (3.5%)	35 (21–45.5)	33 (45.8%)	39 (54.2%)
**Motor neurone disease**	68 (3.3%)	55 (41.75–66.25)	37 (55.2%)	30 (44.8%)
**Parkinson's disease**	61 (3%)	62 (53–73)	40 (65.6%)	21 (34.4%)
**Functional neurological disorders**	48 (2.4%)	20 (17–23.25)	14 (29.2%)	34 (70.8%)
**Cerebellar disorders**	39 (1.9%)	49 (36–58)	21 (53.8%)	18 (46.2%)
**Headache**	35 (1.7%)	33 (24–39.5)	15 (42.9%)	20 (57.1%)
**Dementia**	20 (1%)	67 (59–73.25)	11 (55%)	9 (45%)
**Demyelinating disease**	15 (0.7%)	19 (16–29.5)	6 (40%)	9 (60%)

**Footnote** *missing values, ages=20, sex=4.

The most frequent neurological disorder was cerebrovascular disease 19.9%, followed by paraplegia 13.6%. Taken together as an infection group; meningitis/encephalitis, brain abscess, cerebral malaria, tetanus, and others made up 13.6% of patients. A wide range of other neurological disorders were well represented including peripheral neuropathies 8%, brain tumours 7.9%, seizure disorders 6.4%. cranial nerve palsies 4.8%, movement disorders 4.4%, Parkinson's disease 3%, altered level of consciousness 3.6%, neuromuscular disorders 3.5%, motor neurone disease 3.3%, functional disorders 2.4% and cerebellar disease 1.9%. The frequency of headache 1.7%, dementia1%, and demyelinating disorders 0.7% were low. The median age of these groups varied with disorder, with dementia 67 years, (IQR 59–73.25) Parkinson's disease 62 years, (IQR 53–73) and motor neu ron disease 55 years, (IQR 41.75–66.25) affecting older age groups.

Patients with infectious CNS causes tended to have a lower median age of presentation ranging from 33 years, (IQR 25–40) in meningitis to 37 years, (IQR 29.5–45) in intracerebral abscess. Other groups with younger median ages of presentation included demyelinating disease 19 years, (IQR 16–29.5) functional disorders 20 years, (IQR 17–23.25) and neuromuscular disorders 35 years, (IQR 21–45.5).

**Figure 1:**
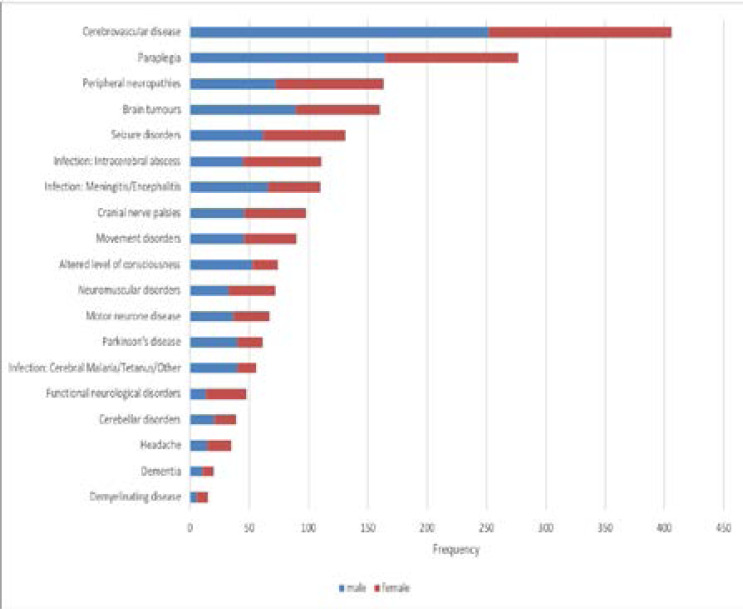
Neurological Disorders by sex *(n=2037)*.

The pattern of main neurological disorders by sex is outlined in Figure 1. The most notable differences in gender distribution was a predominance of males presenting with cerebrovascular disease 62.1%, Parkinson's disease 65.6%, meningitis/encephalitis 60%, tetanus 85.0% and paraplegia 59.6% Females were more frequently represented in functional disorders 70.8%, intracerebral abscess 59.5%, headache 57.1% and peripheral neuropathy 55.2%.

The pattern of neurological disorders in a selected outpatient cohort are presented in Figure 2

The results for the analysis of the association of HIV with neurological disorders is presented in Table 3. Following adjustment for age and sex and in those tested for HIV a wide range of groups of NDs showed an increased odds of HIV when compared to the baseline group of brain tumours. Intracerebral abscesses were most strongly associated with HIV infection (aOR 107, 95% CI 35.1–470.7), followed by meningitis/encephalitis (aOR 40.1, 95% CI 13.6–172.9).

**Table 3:** Neurological disorders and their association with HIV (n=2037)

Neurological diagnosis	Tested for HIV (%)	HIV positive (%)	Odds ratio for HIV positive (95% CI), n=973	Adjusted odds ratio for HIV positive * (95% CI), n=958	Adjusted p-value
**Brain tumours**	83/161 (51.6%)	3 (3.6%)	1 (-)	1 (-)	0.00
**Cerebrovascular disease**	145/406 (35.7%)	26 (17.9%)	5.8 (2–25)	5.6 (1.9–23.9)	0.01
**Infection: Intracerebral abscess**	105/111 (94.6%)	84 (80%)	106.7 (35.4–465.6)	107 (35.1–470.7)	0.00
**Infection: Meningitis/Encephalitis**	98/110 (89.1%)	56 (57.1%)	35.6 (12.2–151.9)	40.1 (13.6–172.9)	0.00
**Infection: Cerebral** **Malaria/Tetanus/Others**	33/57 (57.9%)	9 (27.3%)	10 (2.7–47.8)	11.1 (3–53.6)	0.00
**Paraplegia**	131/278 (47.1%)	25 (19.1%)	6.3 (2.1–27.1)	6.2 (2.1–26.9)	0.00
**Peripheral neuropathies**	90/163 (55.2%)	27 (30%)	11.4 (3.8–49.4)	10.5 (3.5–45.7)	0.00
**Seizure disorders**	39/131 (29.8%)	15 (38.5%)	16.7 (5–76.4)	15.7 (4.6–73)	0.00
**Cranial nerve palsies**	62/98 (63.3%)	22 (35.5%)	14.7 (4.7–64.6)	13.9 (4.4–61.3)	0.00
**Movement disorders**	29/90 (32.2%)	9 (31%)	12 (3.3–57.9)	11.2 (3–54.3)	0.00
**Altered level of consciousness**	34/74 (45.9%)	3 (8.8%)	2.6 (0.5–14.6)	2.9 (0.5–16.8)	0.20
**Neuromuscular disorders**	40/72 (55.6%)	3 (7.5%)	2.2 (0.4–12.2)	2.1 (0.4–11.8)	0.39
**Motor neurone disease**	35/68 (51.5%)	6 (17.1%)	5.5 (1.4–27.5)	4.8 (1.2–24.3)	0.03
**Parkinson's disease**	9/61 (14.8%)	1 (11.1%)	3.3 (0.2–29.8)	3 (0.1–27.4)	0.37
**Functional neurological disorders**	8/48 (16.7%)	1 (12.5%)	3.8 (0.2–34.7)	4.7 (0.2–43.6)	0.21
**Cerebellar disorders**	21/39 (53.8%)	12 (57.1%)	35.6 (9.4–179.9)	33.9 (8.9–172.3)	0.00
**Headache**	11/35 (31.4%)	4 (36.4%)	15.2 (2.8–92)	14.4 (2.7–88.7)	0.00
**Dementia**	7/20 (35%)	0 (0%)	0 (0–0)	0 (0–0)	0.99
**Demyelinating disease**	12/15 (80%)	0 (0%)	0 (0–0)	0 (0–0)	0.98

**Footnote:** Includes logistic regression odds ratio and adjusted odds ratio (a OR), adjusted for age and sex compared to the baseline group of space occupying lesions. The p-value for the Wald test for each category is also presented.

* missing values, ages=20, sex=4.

These groups also had the highest rates of HIV testing; 105/111 (94.6%) and 98/110 (89.1%) respectively. Largely non-infectious disorders also had higher odds of HIV infection including cerebellar disorders (aOR 33.9, 95% CI 8.9–172.3), peripheral neuropathies (aOR 10.5, 95% CI 3.5–45.7), and cerebrovascular disease (aOR 5.6, 95% CI 1.9–23.9). Testing rates were lower in these groups, particularly cerebrovascular disease in whom 145/406 (35.7%) were tested.

There were differences between those who were and were not tested for HIV. The mean age for those tested was younger at 39.9 years, compared to 48.5 years for those not tested (t test statistic 10.4, p<0.05). The unadjusted odds ratio of being tested for HIV in men compared to women was 0.81, 95% CI 0.68–0.97 (p = 0.02); suggesting that men were 19% less likely to be tested for HIV than women.

## Discussion

This study reports a large series of adult patients presenting with NDs and their association with HIV infection. The most common NDs in the series were cerebrovascular disease, infections and paraplegia followed by neuropathies, space occupying lesions tumours and seizures, a pattern largely similar to that reported in other hospital based studies in SSA^3^–^5^. In the outpatient series, (supplementary Figure 1) Figure 2, the most common NDs were peripheral neuropathies, cranial nerve palsies, seizure disorders, movement disorders, paraplegia and motor neurone disease, a pattern also similar to other studies in SSA^4^, ^9^–^11^, ^16^. The low median age of 43 years reported in the overall series is likely a reflection of the relatively low life expectancy of around 60 years in Tanzania during the time of the study, with infections, seizures, neuromuscular disorders, demyelinating disorders, functional disorders, and headache affecting younger age groups and cerebrovascular disease, motor neurone disease and dementia characteristically affecting older age groups. Males were more frequently represented in cerebrovascular disease, meningitis, paraplegia, Parkinson's disease, and tetanus whereas females were more frequently represented in intracerebral abscess, peripheral neuropathies, functional neurological disorders, and headache.

The results of HIV testing were available for 48.7% of patients of whom 30.6% were seropositive. Similar high rates of HIV seropositivity, 15.4–33.3% have been reported previously in patients presenting with NDs in SSA^3^–^5^. NDs in this series significantly associated with HIV included cerebrovascular disease, infections, paraplegia, peripheral neuropathies, cranial nerve palsies, seizure disorders, movement disorders, motor neurone disease, cerebellar disorders, and headache with adjusted odds ratio rangingfrom 107 in cerebral abscess to 4.8 in motor neurone disease.

The finding of a lower mean age of 39.9 years in those screened for HIV infection versus 48.5 years in those not screened and with females more likely to be screened than males suggests there was a clinical selection bias towards screening patients who were more likely to be seropositive. A significant limitation in the present study is that HIV screening was carried out in less than half of all patients and there may have been a selection bias towards referral and inclusion of seropositive patients which may have altered results. An earlier study carried out at the same study site during the same study period involved HIV screening in a consecutive series of 337 adult patients presenting with neurological disorders reported a lower overall HIV seroprevalence of 20.5% with significant HIV association limited to CNS infections^5^.

Cerebrovascular disease was the most common ND in the series with stroke accounting for 14%. Stroke is the second leading cause of death globally^1^, ^12^ and the leading neurological cause of hospital admissions and neurological cause of death in adults in SSA^5^, ^7^. In the series cerebrovascular disease was significantly associated with HIV, OR 5. HIV is known to be an independent risk factor for stroke, predominantly ischaemic, and a similar pattern and the association with HIV has been reported in SSA^5^, ^15^, ^17^, ^18^. Some patients in the series who presented clinically with a stroke like episode were found on investigation to have other forms of cerebrovascular disease including chronic subdural haematoma (SDH) 2.9% and subarachnoid haemorrhage (SAH)1.2%. Patients with SDH were more likely to be male, (73.3%) and older, (median age 62 years). They typically presented without a clear history of head injury whose diagnosis was established on neuroimaging. The series has been reported elsewhere in a recent publication^19^. In contrast patients presenting with SAH were more likely to be female, (76%) and younger, (median age 38 years).

Infections accounted for 13.6% in the series and were strongly associated with HIV infection with OR ranging from 107 in cerebral abscess to 40.1 in meningitis. The association with cerebral abscess was strongest for toxoplasmosis and for the category toxoplasmosis / tuberculosis/cryptococcus where a precise aetiology could not be firmly established. In contrast there was no association of pyogenic brain abscess with HIV. The association of HIV with meningitis was strongest for cryptococcal meningitis and also for tuberculous and acute bacterial meningitis. The pattern of CNS opportunistic infections (OIs) in HIV disease reported in this series is similar to that reported elsewhere in SSA^15^. OIs in HIV infection involving the CNS are known to occur in as many as 20% of HIV infected persons and are responsible for over 20% of HIV related deaths in SSA^20^–^24^. Other infections reported in the series which were not associated with HIV infection included cerebral malaria 0.8% and tetanus 1.1%.

Non Traumatic paraplegia (PP) accounted for 13.6% in the series and was significantly associated with HIV infection, OR 6.2. The main causes in the series were malignancy, transverse myelitis and Potts disease, with a group (35%) of undetermined aetiology, a pattern largely similar to that reported in other studies in Africa^25^. The association of paraplegia and HIV infection has been well documented in SSA, affecting a mostly younger age group (20–40 years) and with TB, syphilis and vacuolar myelopathy as the main causes^15^, ^26^–^28^. A small number of patients in the series 0.7%, presented with central demyelinating neurological disorders, including neuromyelitis optica (NMO), acute demyelinating encephalomyelitis (ADEM) and multiple sclerosis (MS), and without an association with HIV. However NMO has been reported in a large series from South Africa with a significant association with HIV^29^. Multiple sclerosis is relatively uncommon in Africa and was reported in only two patients in the present series. In a detailed study in South Africa the age standardized prevalence rate of MS was reported as 0.23/100 000 in the black population was as compared to 25.64/100,00 in the white population^30^.

Peripheral neuropathies accounted for 8% in the series and was associated with HIV infection, OR 10.5. Neuropathy has been reported in other neurology studies in SSA with frequencies ranging from 10% in inpatients series and 2.2–13.8% in outpatients series^4^, ^9^, ^10^, ^16^, ^31^. The most common neuropathy in HIV in SSA is distal sensory neuropathy with reported frequencies in HIV in SSA ranging from 4–64% largely depending on diagnostic criteria used, including HIV stage and antiretroviral therapy^32^–^35^. While the present series included selected referred patients with Guillain-Barré syndrome (GBS), hereditary disorders and subacute combined degeneration, in the majority of patients the neuropathies were classified as either secondary to systemic disease or idiopathic.

GBS was reported in 1.3% of patients with a HIV seropositive rate of 26.3% in those screened. The association of inflammatory neuropathies with HIV infection has been reported in SSA since early in the epidemic^36^–^38^. Cranial nerve palsies accounted for 4.8% of NDs in the series and were associated with HIV infection, OR 13.9. The association was strongest for facial nerve palsy and optic neuritis, a pattern previously reported in associated with HIV infection in SSA^39^, ^40^.

Movement disorders 4.4%, and Parkinson's disease 3%, which was categorised separately, together accounted for a total of 7.4% of NDs in the series. The category movement disorders included dystonia, chorea, benign essential tremor and writer's cramp, a pattern largely similar to that reported elsewhere in SSA^41^, ^42^. In this series movement disorders were significantly associated with HIV infection, OR 11.2. While movement disorders in HIV infection may occur as a result of CNS opportunistic infections they may also occur as a result of the direct effects of HIV infection involving the brain^15^. Parkinson's disease in the series was not associated with HIV. It typically affected mostly older males, a pattern similar to that reported elsewhere in SSA^43^–^45^.

Brain tumours accounted for 7.9% of NDs in the series and had a low overall HIV prevalence rate of 3.6%. The main pattern, metastatic, glioma, meningioma and pituitary tumours is similar to that reported in other series in Africa. The frequency of primary CNS lymphoma, the most common brain tumour in HIV is known to be low in SSA where the confirmed autopsy rate ranges from 0 to 1.3%^21^.

Neuromuscular disorders accounted for 3.5% of NDs in the series and were not significantly associated with HIV, OR 2.1. They comprised of myasthenia gravis, dermatomyositis / polymyositis, stiff person syndrome, muscular dystrophy, and others. An association of dermatomyositis/polymyositis with HIV has been reported in SSA^46^. Stiff person syndrome is a very rare neuromuscular disorder and a more detailed analysis of the cases reported in the present case series has been published elsewhere^47^. The neuromuscular disease category included limb girdle and fascio scapula humeral muscular dystrophy and the first genetically confirmed case of Becker muscular dystrophy in Tanzania^48^.

Motor neuron disease (MND) comprised 3.3% in the series and was significantly associated with HIV infection, OR 4.8. A recent publication from Tanzania involving 116 MND patients including patients from the present series describes the full clinical spectrum of MND and their significant association with HIV. It also includes a brief report of a patient who presented with symptomatic HIV and new onset concurrent amyotrophic lateral sclerosis and was treated with antiretroviral treatment (ART) and survived for over 10 years^49^. The association between HIV and MND is more strongly confirmed in a recent study from South Africa involving a cohort of 35 HIV positive MND patients with a similar reported 10 year survival of 48% in the ART treated cohort versus 3% in the HIV uninfected MND cohort^50^. This novel association of MND with HIV and apparent response of MND to ART is reviewed in a recent editorial suggesting the reactivation of latent retroviral infection as the likely aetiological agent in HIV associated MND^51^.

Seizure disorders comprised 6.4% of NDs in the series and were significantly associated with HIV infection, OR 15.7. A high burden of seizure disorders with frequencies ranging from 6–12.6% is reported in similar hospital based studies in adults in SSA^3^, ^5^, ^52^. The added burden of seizures in HIV in SSA is reflected by their reported high frequency in HIV infection, occurring in up to 10% of patients presenting with advanced immunosuppression, with OIs as their main cause^5^, ^15^, ^53^, ^54^. Impaired level of consciousness was present in 3.6% in the series and was not associated with HIV infection, OR 2.9. It is associated with a poor prognosis in SSA, with reported in hospital case fatality rates ranging from 26–76% typically reflecting the seriousness of the underlying cause.^5^, ^55^–^57^.

Cerebellar disorders accounted for 1.9% of NDs in the series including hereditary and idiopathic and were significantly associated with HIV infection, OR 33.9. The main causes of cerebellar disease in HIV are focal opportunistic processes involving the cerebellum including toxoplasmosis and progressive multifocal leukoencephalopathy and less frequently CNS lymphoma 58. However less well known are chronic effects of direct HIV infection which can result in cerebellar atrophy and clinical disease^59^.

Functional NDs accounted for 2.4% in the series and were characterised by a younger age group affected and female preponderance but without any association with HIV infection, OR 3.8. The main pattern of clinical presentations included loss of neurological function, psychogenic non-epileptic attacks, and movement disorders. A more detailed report of the patients reported in the present series and a review of the literature has been recently published 60.

Headache accounting for 1.7% in the series included migraine, cluster headache and idiopathic intracranial hypertension and were significantly associated with HIV infection, OR 14.4. However the sample size reported is small and an underestimate of their true burden with most cases either not referred for neurological consultation or reported. The burden of headache globally is known to be large affecting as many as 3 billion persons, with almost 2 billion with tension headache and1 billion with migraine 61 Studies in SSA suggest that the burden and pattern of headache is largely similar to that reported in high income countries^62^–^64^. A small series of predominantly male patients presented with cluster headache and while it has been reported in SSA it appears to be relatively uncommon there^65^. Idiopathic intracranial hypertension occurred almost exclusively in younger, (median age 28.5 years), females (90%) and has been reported elsewhere in SSA^66^.

Dementia represented just 1% of NDs in this series study and characteristically affected an older age group, (median age 67 years) and was not associated with HIV. The low frequency of dementia reported in the present series is likely to be a significant underestimate of the true burden as most cases were either not recognized, referred, or reported. The Global Burden of dementia is large with an age standardized prevalence rate of 712/100,000 and an estimated global burden of almost 44 million cases^67^. Dementia is reported in neurology studies in SSA as occurring with a wide range of frequencies ranging from 0–11%^68^, ^69^. An age standardized community prevalence in those aged 70 and over in Northern Tanzania, none of whom had been previously diagnosed, was reported as 6.4% (CI 4.9–7.9), a frequency comparable to that reported in HICs^70^. While the main causes of dementia are Alzheimer's and cerebrovascular disease, the added burden of HIV associated neurocognitive disorders (HAND) coupled with an increasingly aging population makes it likely that SSA will experience a high frequency of dementia in the future^71^.

The outcome is not reported in this case series. However an earlier study involving a consecutive series of 337 adult neurology inpatients at the same study site and during the same study period reported that the outcome at discharge was death 27.6%, disability 54%, and no disability 18.4%, with death, 39.1% more likely in seropositive patients^5^. Case fatality rates were highest for tetanus 71.4%, meningitis 57.1%, cerebral malaria 42.9% and other CNS infections 37.1%. Patients with stroke, paraplegia and space occupying lesions were more likely to be discharged with disability.

## Limitations

This paper has some limitations. There is likely to be selection bias as the series represents tertiary referrals to a specialist neurology service, the only such service serving 16 million persons. It is likely biased towards the more uncommon neurological diseases, such as motor neurone disease and space occupying lesion and diagnoses which benefit from specialist opinion and diagnostics such as neuroimaging. Conversely, the neurological diseases which local services are more used to managing independently such headache, and diseases which may not present due to reduced awareness, such as dementia, are less well represented in the series.

This study does not aim to differentiate between inpatient and outpatients but rather present an overview of a tertiary service. There is likely to be differences in these groups which would merit further and more detailed investigation. Rates of HIV testing differed by disorders and the epidemiological characteristics of those tested differed from those not tested. The adjusted odds ratios of HIV infection should therefore be interpreted with significant caution as a differential bias may have occurred. Nevertheless, broadly rates of HIV infection appear high and the significant odds ratio between not only opportunistic infections, but also in other NDs conditions such as stroke and paraplegia support a conclusion that HIV is associated with these diseases.

Finally, given the relative absence of more detailed laboratory and neuroradiological diagnostic facilities, and sub-specialty referral there may be concerns regarding the accuracy of diagnosis. The ND/diagnoses categories used represents best clinical practice in the study setting and more detailed clinical characteristics missing in this present study are addressed in the separate published nested study 5.

## Conclusion

This hospital-based study reports a large series of NDs and diseases in adults in Tanzania and describes their main pattern of demographics and categorization. The study reports a significant association of a range of NDs with HIV infection including stroke, infections, paraplegia, neuropathies, cranial nerve palsies, movement disorders, motor neuron disease, seizures, cerebellar disease, and headache. It is likely that HIV will continue to influence the pattern of NDs in SSA for the foreseeable future. Routine screening for HIV is recommended for patients presenting with NDs.

## Figures and Tables

**Table 4:** Neurological disorder and diagnosis by age, HIV status and sex (n=2037)

Disorder	Diagnosis	Frequency (%)	*Median age (IQR)	HIV positive (%)	*Male (%)	*Female (%)
**Cerebrovascular** **disease**	Cerebrovascular accident	286 (14%)	56.5 (42–68)	24 (27.3%)	186 (65%)	100 (35%)
	Subdural haematoma	60 (2.9%)	62 (51–76)	0 (0%)	44 (73.3%)	16 (26.7%)
	Subarachnoid haemorrhage	25 (1.2%)	38 (25–53)	2 (16.7%)	6 (24%)	19 (76%)
	Others	35 (1.7%)	33 (25.5–46)	0 (0%)	16 (45.7%)	19 (54.3%)
**Infection:** **Intracerebral** **abscess**	Toxoplasmosis	47 (2.3%)	37 (30–46)	47 (100%)	15 (31.9%)	32 (68.1%)
	Toxo/Tuberculous/Crypt ococcus	31 (1.5%)	41 (35–45)	30 (100%)	14 (45.2%)	17 (54.8%)
	Pyogenic abscess	22 (1.1%)	35 (21.25–50.5)	1 (5.6%)	11 (50%)	11 (50%)
	Tuberculous	11 (0.5%)	30 (28–36)	6 (60%)	5 (45.5%)	6 (54.5%)
**Infection:** **Meningitis/** **Encephalitis**	TB meningitis	44 (2.2%)	33 (27–39)	16 (39%)	30 (68.2%)	14 (31.8%)
	Cryptococcal meningitis	34 (1.7%)	35 (30–41)	33 (100%)	16 (47.1%)	18 (52.9%)
	Bacterial meningitis	25 (1.2%)	25 (22–38)	6 (33.3%)	16 (64%)	9 (36%)
	Viral meningoencephalitis	7 (0.3%)	38 (19–55)	1 (16.7%)	4 (57.1%)	3 (42.9%)
**Infection: Cerebral** **Malaria/ Tetanus/** **Others**	Tetanus	20 (1%)	39 (25.75–49.75)	0 (0%)	17 (85%)	3 (15%)
	Cerebral Malaria	17 (0.8%)	28 (20–31)	1 (9.1%)	10 (62.5%)	6 (37.5%)
	Others	20 (1%)	35.5 (21.75–56)	8 (50%)	13 (65%)	7 (35%)
**Paraplegia**	Malignancy spinal cord	73 (3.6%)	57 (43–70)	3 (9.1%)	50 (68.5%)	23 (31.5%)
	Paraplegia	65 (3.2%)	45.5 (30–55)	10 (41.7%)	28 (43.75%)	36 (56.25%)
	Transverse myelitis	37 (1.8%)	30 (20–38)	1 (4.5%)	18 (48.6%)	19 (51.4%)
	Quadriplegia	24 (1.2%)	57 (47.5–62.25)	0 (0%)	19 (79.2%)	5 (20.8%)
	Potts Disease	23 (1.1%)	36 (29–51.5)	5 (35.7%)	12 (52.2%)	11 (47.8%)
	Epidural abscess	13 (0.6%)	27 (17–35)	0 (0%)	8 (61.5%)	5 (38.5%)
	Syringomyelia	12 (0.6%)	48 (42–49)	0 (0%)	5 (41.7%)	7 (58.3%)
	Others	31 (1.5%)	34.5 (25.25–51.5)	6 (33.3%)	25 (80.6%)	6 (19.4%)
**Peripheral** **neuropathies**	Idiopathic	47 (2.3%)	38 (28.5–48.5)	5 (18.5%)	21 (44.7%)	26 (55.3%)
	Guillain Barré syndrome	29 (1.4%)	28 (18–44)	5 (26.3%)	12 (41.4%)	17 (58.6%)
	Hereditary Sensory Motor Neuropathies (HMSN)	14 (0.7%)	35 (30.25–53)	3 (37.5%)	8 (57.1%)	6 (42.9%)
	Subacute combined degeneration	12 (0.6%)	44 (37–49.25)	0 (0%)	5 (41.7%)	7 (58.3%)
	Mononeuritis multiplex	10 (0.5%)	38.5 (32.25–42.75)	3 (60%)	5 (50%)	5 (50%)
	Secondary and Others	51 (2.5%)	45 (35.5–55)	11 (45.8%)	22 (43.1%)	29 (56.9%)
**Brain tumours**	Metastatic	80 (3.9%)	45 (34–61)	2 (5.3%)	50 (62.5%)	30 (37.5%)
	Glioma	39 (1.9%)	49 (28.25–62)	0 (0%)	22 (56.4%)	17 (43.6%)
	Meningioma	21 (1%)	41.5 (35–53.5)	1 (6.7%)	7 (35%)	13 (65%)
	Pituitary Tumour	11 (0.5%)	43 (39–49)	0 (0%)	7 (63.6%)	4 (36.4%)
	Others	10 (0.5%)	34.5 (19–46.25)	0 (0%)	4 (40%)	6 (60%)
**Cranial nerve** **palsies**	Facial nerve palsy	36 (1.8%)	33 (25.5–48.5)	7 (25.9%)	21 (58.3%)	15 (41.7%)
	Optic neuritis	21 (1%)	40 (35–46)	11 (61.1%)	5 (23.8%)	16 (76.2%)
	III,IV,VI nerve palsy	15 (0.7%)	50 (36–58.5)	1 (12.5%)	7 (46.7%)	8 (53.3%)
	Trigeminal neuralgia	15 (0.7%)	48 (35.5–64)	2 (66.7%)	7 (46.7%)	8 (53.3%)
	Others	11 (0.5%)	41 (35.25–57)	1 (16.7%)	6 (54.5%)	5 (45.5%)
**Movement** **disorders**	Dystonia	31 (1.5%)	43 (22.5–53)	2 (18.2%)	14 (45.2%)	17 (54.8%)
	Chorea	15 (0.7%)	62 (26.5–69)	1 (33.3%)	8 (53.3%)	7 (46.7%)
	Benign Essential Tremor	13 (0.6%)	67 (57–77)	2 (100%)	8 (61.5%)	5 (38.5%)
	Involuntary Movements	12 (0.6%)	43 (26.5–49.25)	2 (33.3%)	5 (41.7%)	7 (58.3%)
	Others	19 (0.9%)	35 (31–54)	2 (28.6%)	11 (57.9%)	8 (42.1%)
**Neuromuscular** **disorders**	Myasthenia gravis	25 (1.2%)	33 (21–43)	0 (0%)	10 (40%)	15 (60%)
	Dermatomyositis	15 (0.7%)	40 (36–54.5)	1 (10%)	5 (33.3%)	10 (66.7%)
	Stiff person syndrome	9 (0.4%)	45 (37–48)	1 (14.3%)	5 (55.6%)	4 (44.4%)
	Limb girdle dystrophy	6 (0.3%)	24.5 (15.25–30.75)	0 (0%)	5 (83.3%)	1 (16.7%)
	Facio scapulo humeral dystrophy	4 (0.2%)	16.5 (14.5–19.75)	0 (0%)	3 (75%)	1 (25%)
	Others	13 (0.6%)	32 (25–37)	1 (12.5%)	5 (38.5%)	8 (61.5%)
**Motor neurone** **disease**	Amyotrophic lateral sclerosis	42 (2.1%)	50.5 (40.25–63.5)	4 (16.7%)	21 (51.2%)	20 (48.8%)
	Pseudobulbar palsy	10 (0.5%)	70 (56.25–74.5)	0 (0%)	3 (30%)	7 (70%)
	Progressive muscular atrophy	10 (0.5%)	53.5 (49–65.75)	1 (16.7%)	9 (90%)	1 (10%)
	Primary lateral sclerosis	6 (0.3%)	60.5 (53.25–67.75)	1 (33.3%)	4 (66.7%)	2 (33.3%)
**Parkinson's disease**	Parkinson's disease	56 (2.7%)	60 (52.75–70.5)	1 (14.3%)	36 (64.3%)	20 (35.7%)
	PD plus syndrome	5 (0.2%)	74 (71–75)	0 (0%)	4 (80%)	1 (20%)
**Altered state of** **consciousness**	Altered level of consciousness/Coma	47 (2.3%)	40 (25–52.5)	2 (8.7%)	34 (72.3%)	13 (27.7%)
	Overdose	16 (0.8%)	26 (22.25–47.25)	1 (14.3%)	11 (68.8%)	5 (31.3%)
	Other	11 (0.5%)	39.5 (26–45)	0 (0%)	8 (72.7%)	3 (27.3%)
**Functional** **neurological** **disorders**	Functional paralysis	30 (1.5%)	20 (17.25–22)	1 (16.7%)	10 (33.3%)	20 (66.7%)
	Psychogenic non- epileptic attacks	17 (0.8%)	19 (16–30)	0 (0%)	3 (17.6%)	14 (82.4%)
	Globus hystericus	1 (0%)	27 (27–27)	-	1 (100%)	0 (0%)
**Seizure disorders**	Generalised Seizures	114 (5.6%)	33 (21–48)	15 (45.5%)	54 (47.4%)	60 (52.6%)
	Others	17 (0.8%)	27 (17–39)	0 (0%)	8 (47.1%)	9 (52.9%)
**Cerebellar** **disorders**	Cerebellar Ataxia	35 (1.7%)	49 (36.5–58)	12 (57.1%)	18 (51.4%)	17 (48.6%)
	Friedreich's ataxia	4 (0.2%)	32 (21.75–45.75)	-	3 (75%)	1 (25%)
**Headache**	Idiopathic intracranial hypertension	10 (0.5%)	28.5 (24.5–38.25)	1 (20%)	1 (10%)	9 (90%)
	Cluster headache	9 (0.4%)	38 (33–41)	-	8 (88.9%)	1 (11.1%)
	Migraine	8 (0.4%)	30 (20.25–38.5)	0 (0%)	2 (25%)	6 (75%)
	Others	8 (0.4%)	27.5 (21–31.75)	3 (75%)	4 (50%)	4 (50%)
**Dementia**	Dementia	13 (0.6%)	67 (59–70)	0 (0%)	7 (53.8%)	6 (46.2%)
	Frontal lobe	5 (0.2%)	60 (56–72)	0 (0%)	3 (60%)	2 (40%)
	Others	2 (0.1%)	70 (60–79)	-	1 (50%)	1 (50%)
**Demyelinating** **disease**	Neuromyelitis Optica	9 (0.4%)	25 (19–30)	0 (0%)	3 (33.3%)	6 (66.7%)
	Acute demyelinating encephalomyelitis	4 (0.2%)	15.5 (13–18.75)	0 (0%)	2 (50%)	2 (50%)
	Multiple Sclerosis	2 (0.1%)	21 (17–25)	0 (0%)	1 (50%)	1 (50%)

**Footnote** *missing values, ages=20, sex=4

## Abbreviations

ADEM: Acute Demyelinating Encephalomyelitis; aOR: Adjusted Odds Ratio; ART: Antiretroviral treatment; CNS: Central Nervous System; CI: Confidence Intervals; CT: Computerized Tomography; DALYS: Disability-Adjusted Life Years; DSN: Distal Sensory Neuropathy; GBS: Guillain-Barré syndrome; HAND: HIV Associated Neurocognitive Dysfunction; HIV: Human Immunodeficiency Virus; IQR: Interquartile range; KCMC: Kilimanjaro Christian Medical Centre; MND: Motor Neurone Disease. MRI: Magnetic Resonance Imaging; MS: Multiple Sclerosis; ND: Neurological Disorders; NMO: Neuromyelitis Optica; NCD: Non-communicable Diseases; OI: Opportunistic infection; SAH: Sub Arachnoid Haemorrhage; SDH: Subdural Haematoma; SSA: Sub-Saharan Africa; VM: Vacuolar Myelopathy.
